# Body Mass Index of Young Men in China: Results From Four National Surveys Conducted Between 1955 and 2012

**DOI:** 10.1097/MD.0000000000002829

**Published:** 2016-02-12

**Authors:** Yi Wan, Xun Jiang, Yanan He, Yuhai Zhang, Ying Liang, Feng Pan, Yongyong Xu, Lei Shang

**Affiliations:** From the Department of Health Statistics (YW, YH, YZ, YL, FP, YX, LS); Department of Health Service, School of Public Health (YW); and Department of Paediatrics, Tangdu Hospital, Fourth Military Medical University, Xi’an, Shaanxi, China (XJ).

## Abstract

To analyze the characteristics and trends of body mass index (BMI) among young men in China using data from a series of national surveys conducted between 1955 and 2012, and to provide evidence for policy making and disease control and prevention.

BMI-related data were collected by routine medical examination from young men, most aged 18 to 20 years, in 4 national surveys (1955, 1974, 2001, and 2012) using a stratified cluster sampling method in 31 provinces, autonomous regions, and municipalities in China. The characteristics and trends of BMI during this period were analyzed by region, year, age, and economic level.

Totals of 266,791, 118,092, 69,776, and 57,969 participants were included in the 4 national surveys, respectively. Between 1955 and 2012, height, weight, and BMI showed increasing trends in men aged 18 to 20 years at the national level and in each of the 6 areas of China. BMI also differed among geographical regions. Data from the 2012 national survey showed that age (17–22 years) was correlated positively with the prevalence of overweight and negatively with the prevalence of underweight (both *P* < 0.05). Gross domestic product was correlated negatively with the prevalence of underweight (*r* = –0.25) and positively with the prevalence of overweight and obesity (*r* = 0.45 and 0.240, respectively; all *P* < 0.001).

BMI increased with economic development among young men from 1955 to 2012, with distinct variation among geographic areas in China. Although underweight remains prevalent in young men, especially in urban and northern regions, overweight and obesity are increasingly prevalent and warrant public health attention.

## INTRODUCTION

The prevalence of obesity has increased worldwide over the past 30 years.^1,2^ Observational epidemiologic studies have revealed a “U”- or “J”-shaped relationship between body mass index (BMI) and mortality,^3^ with mortality rates elevated in persons with low (<23 kg/m^2^) and high (>28 kg/m^2^) BMI.^3–5^ Most studies have focused on the prevalence of high BMI, associating it with increased risk of conditions such as coronary heart disease, stroke, diabetes, and cancer.^6^ Relative to normal weight, obesity has also been associated with significantly higher all-cause mortality.^7,8^ In 2010, overweight and obesity were estimated to cause 3.4 million deaths, 4% loss of years of life, and 4% loss of disability-adjusted life-years worldwide.^9^ Obesity has become a major global health challenge, and its high incidence will affect future population health and economics.^10^ In the past 3 decades, no nation has been able to successfully control this health problem.^11^

The prevalence of overweight and obesity is increasing rapidly with development in China, especially among youth.^12^ According to a national nutritional survey, 17.4% of the Chinese population was overweight in 1992, and the government's key agenda focused on the prevalence of underweight individuals. In 2002, the same survey showed that 29% of individuals were overweight, with a large (66.7%) increase in the prevalence of overweight and obesity.^13^

Up-to-date information about BMI levels and trends is essential to quantify health effects and to prompt decision makers to prioritize action and assess the progress of measures.^11^ This study aimed to analyze BMI characteristics, trends, and their relationship to economic level among young men in China during the period of 1955 to 2012 using data from a series of national surveys, to provide evidence for disease control and prevention and highlight important implications for policy makers.

## METHODS

### Data Source and Participants

Candidates for military service were recruited for 4 national surveys conducted in 1955, 1974, 2001, and 2012 in China. These surveys employed a stratified cluster sampling method to select locations among 2861 counties or cantons in China.^14^ According to population, geography, and economy, several counties or cantons in each selected province were designated as sampling locations. This strategy resulted in coverage of all provinces, autonomous regions, and municipalities in China except the Hong Kong Special Administrative Region, Macao Special Administrative Region, and Taiwan. All recruited youths in each location were surveyed.

The 6 areas of China covered by the surveys are the northeastern area, including Liaoning, Jilin, and Heilongjiang provinces; the northern area, including Beijing and Tianjin cities, Shanxi and Hebei provinces, and the Inner Mongolia autonomous region; the eastern area, including Shanghai city and Jiangsu, Zhejiang, Anhui, Jiangxi, Fujian, and Shandong provinces; the southern-central area, including Hubei, Hunan, Guangdong, Henan, and Hainan provinces; the northwestern area, including Shaanxi, Gansu, and Qinhai provinces and the Ningxia Hui and Xinjiang Uighur autonomous regions; and the southwestern area, including Sichuan, Guizhou, and Yunnan provinces, Chongqing city, and the Guangxi Chuang and Tibet autonomous regions.

Participants were excluded from the study if they were of minority (non-Han) nationality, female, or disabled, or if they had chronic gastroenteritic diseases, endocrinopathy, or other diseases that could cause abnormal weight reduction or gain. Except for the analysis of the relationship between age and BMI using 2012 data, participants aged <18 years and >20 years were also excluded from the analysis to make the age range concordant with the 1955 and 1974 surveys for comparison purposes. The Ethics Committee of the Fourth Military Medical University approved this study, and all participants provided informed consent prior to study participation.

The survey staff included medical researchers, physicians, nurses, and technicians who were trained together using a uniform training document before the survey. In each survey location, each staff member was assigned the responsibility for the measurement or examination of a specific item.^14^ All measurement data from physical examinations were recorded in the field and ultimately transferred to the study center.

### Anthropometric Indicators

Participants’ height and weight were measured using standard methods,^15^ and all task groups used the same precision instruments. A metal column height gauge (200 cm long, 0.1 cm precision) was used to measure height. Participants were required to stand barefooted, with their arms straight and relaxed by their sides, on the measuring instrument. Their heels, buttocks, and scapulae were in contact with the column, and their heads were held in the Frankfort plane, when the distance from the top of the scale to the feet was being measured.

A beam scale (120 kg weight, 0.1 kg precision) was used to measure weight. Participants were required to wear only shorts during the measurement. The instrument was calibrated before measurement and during the measurement period, and the observer checked its accuracy and sensitivity daily.

The World Health Organization's internationally accepted recommended BMI cut-off values for adults were used: underweight, BMI <18.5 kg/m^2^; normal weight, BMI 18.5 to 25.0 kg/m^2^; overweight, BMI 25.0 to 30.0 kg/m^2^; and obese, BMI ≥30.0 kg/m^2^.^16^

### Gross Domestic Product

To address the relationship between BMI and gross domestic product (GDP), data on average GDP per person in each province or autonomous region and municipality were obtained from China's statistical yearbook.^17^

### Statistical Analysis

EpiData 3.1 (The EpiData Association, Odense, Denmark) was used for data entry and management, and SPSS 17.0 for Windows (SPSS Inc., Chicago, IL) was used for statistical analyses.

As only means and standard deviations of height and weight were available from the 1955 and 1974 surveys, the means and variances of BMI were calculated using the Pearson conversion formula:^18^ 
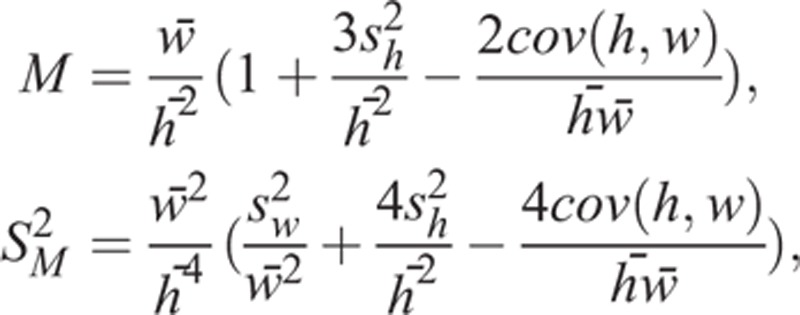


where  
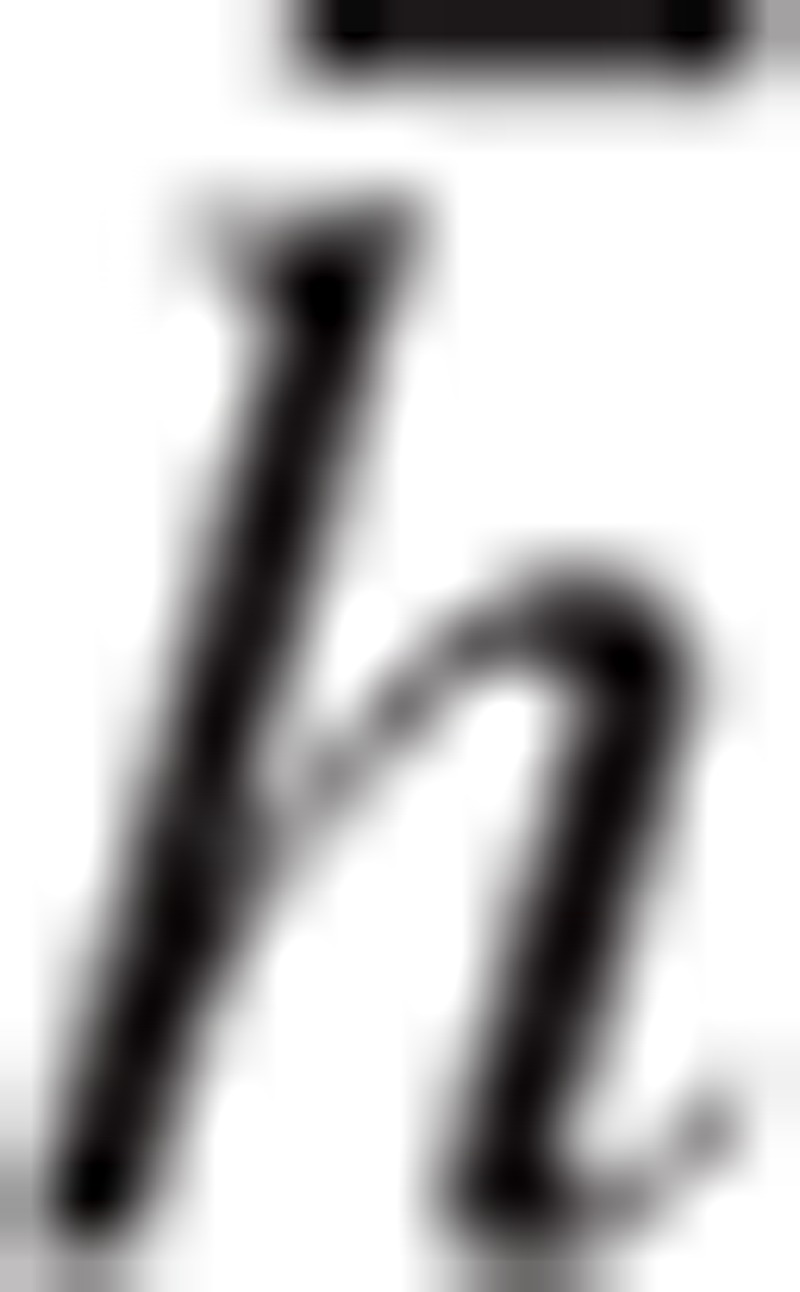
 and  
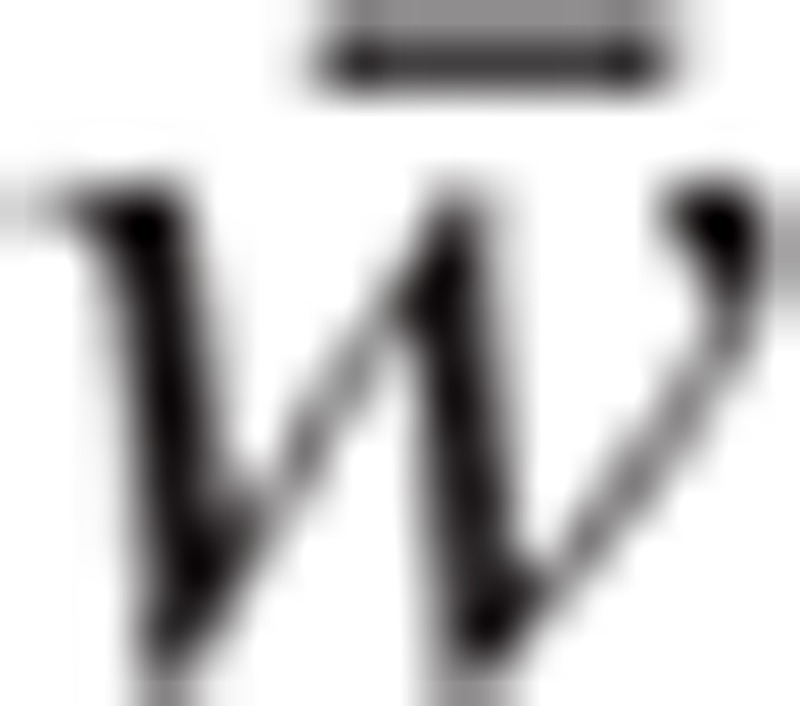
 are the means of height and weight, S_h_ and S_w_ the standard deviations of height and weight, and cov(h,w) is the covariance of height and weight.

We adjusted the proportions of subjects’ ages, which differed significantly among areas, according to the age proportions of all subjects: 48.77% of subjects were aged 18 years, 30.03% were aged 19 years, and 21.19% were aged 20 years. Pearson correlation was used to analyze the relationship between BMI and age/GDP after weighting by the population average. *P* < 0.05 was taken to indicate statistical significance.

## RESULTS

In 1955, a total of 270,959 participants were recruited in all 1301 counties of China. In 1974, data were collected from a total of 139,929 candidates in 120 selected counties. Raw survey data from the 1974 and 1955 surveys were not available due to the passage of time; instead, data from these 2 surveys were taken from statistical reports.^19,20^ In 2001, 100 survey points (counties or cities) were selected and data were collected from a total of 81,193 candidates. In 2012, 101 survey points from 30 provinces were included, and data were collected from 58,076 young male candidates. In total, data from 266,791, 118,092, 69,776, and 57,969 participants in the 4 national surveys, respectively, were included in the present study (Table 1).

**TABLE 1:**
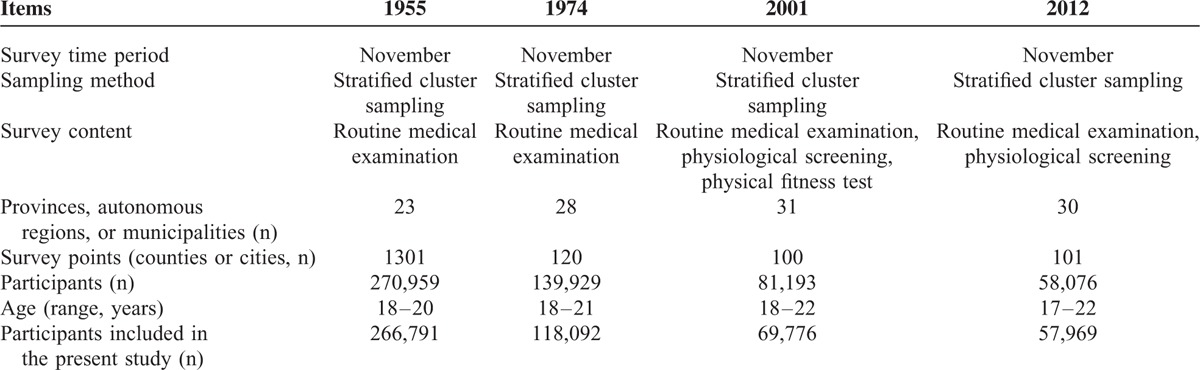
Characteristics of the 4 National Surveys Among Young Men in China

**FIGURE 1:**
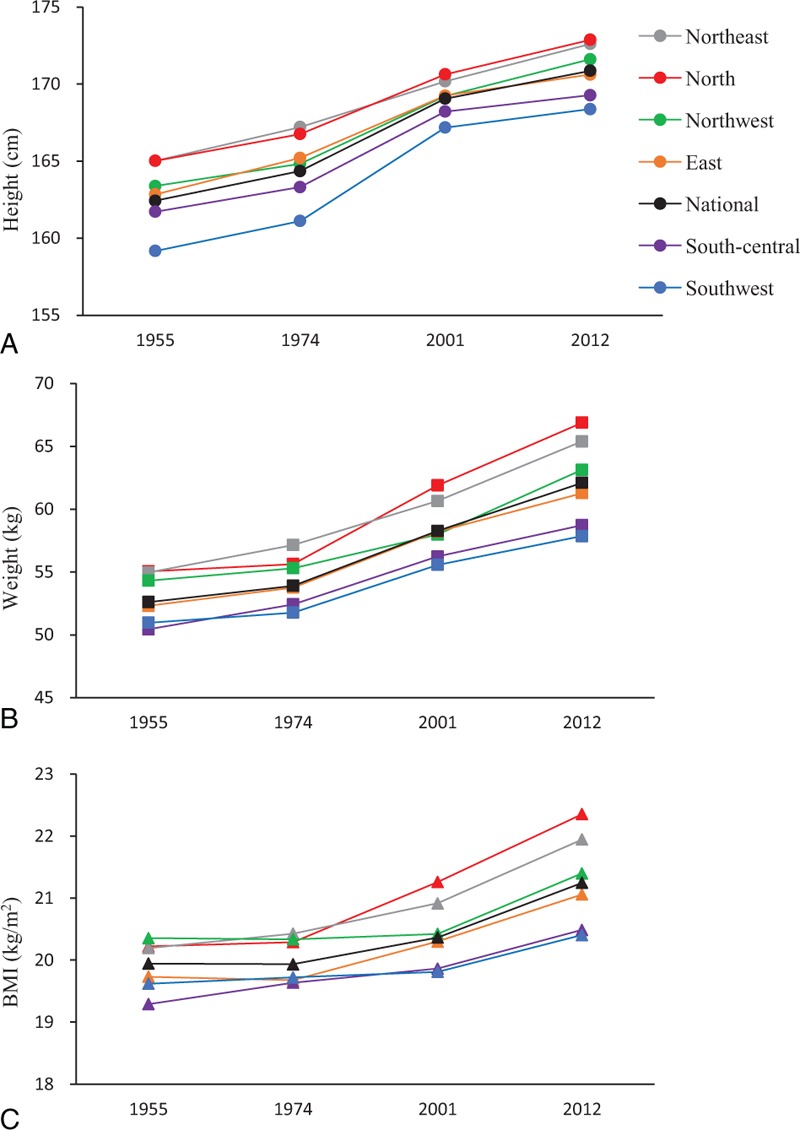
Height (A), weight (B), and body mass index (BMI) (C) among men aged 18 to 20 years in 6 areas of China and at the national level. Data were obtained from 4 national surveys conducted between 1955 and 2012.

**FIGURE 2:**
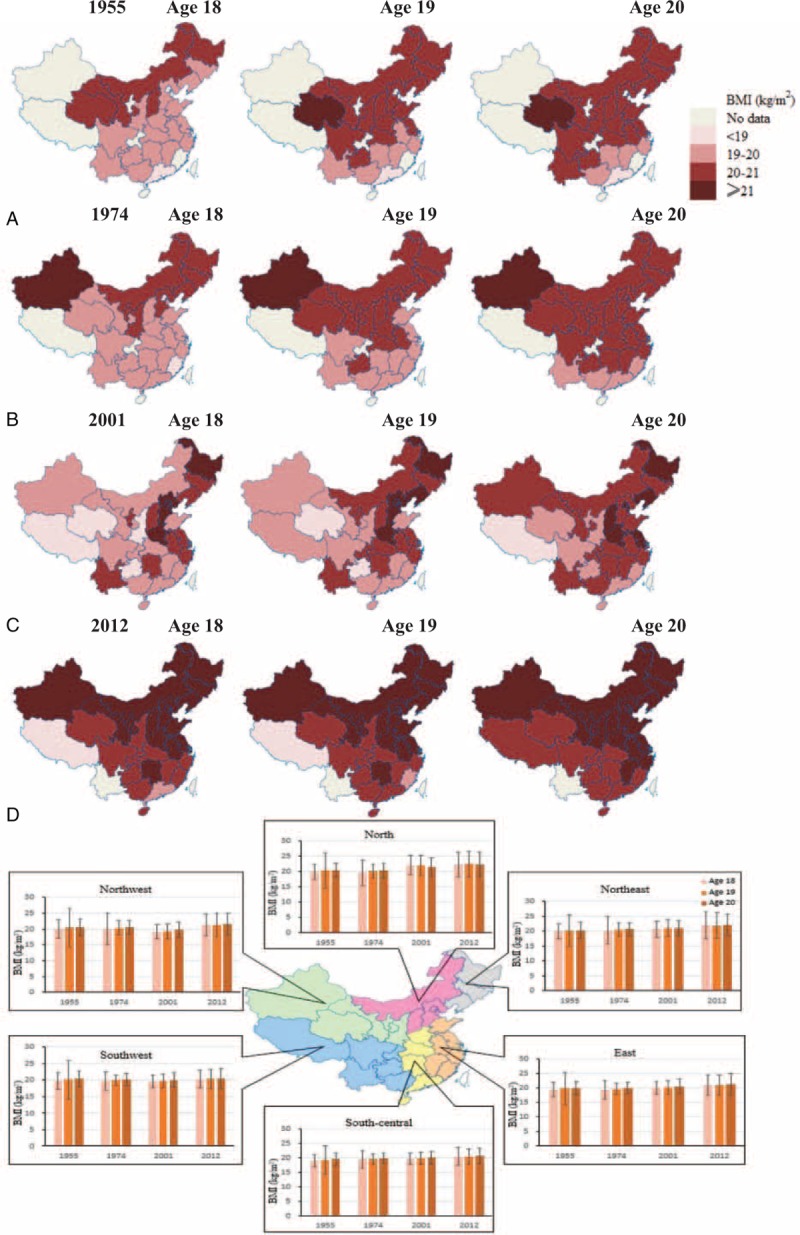
BMI among men aged 18 to 20 years in 6 geographic regions of China. Data  
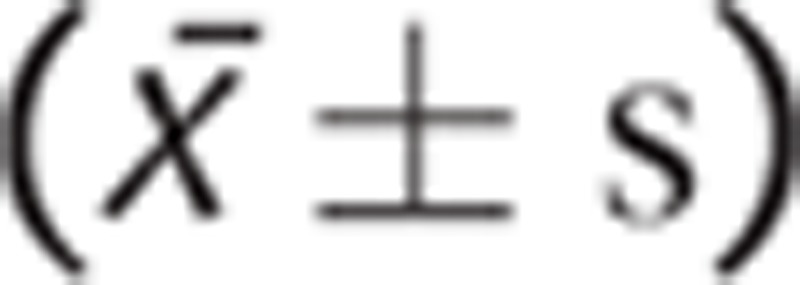
 were obtained from 4 national surveys conducted between 1955 and 2012.

Between 1955 and 2012, height, weight, and BMI showed increasing trends among young men at the national level and in each of the 6 areas of China. BMI differed among geographic regions; it was higher in the northeastern, northern, and northwestern areas, and lower in the southwestern, south-central, and eastern areas. BMI also showed an increasing trend with age, with geographical differences (all *P* < 0.05; Figures 1–2).

**FIGURE 3:**
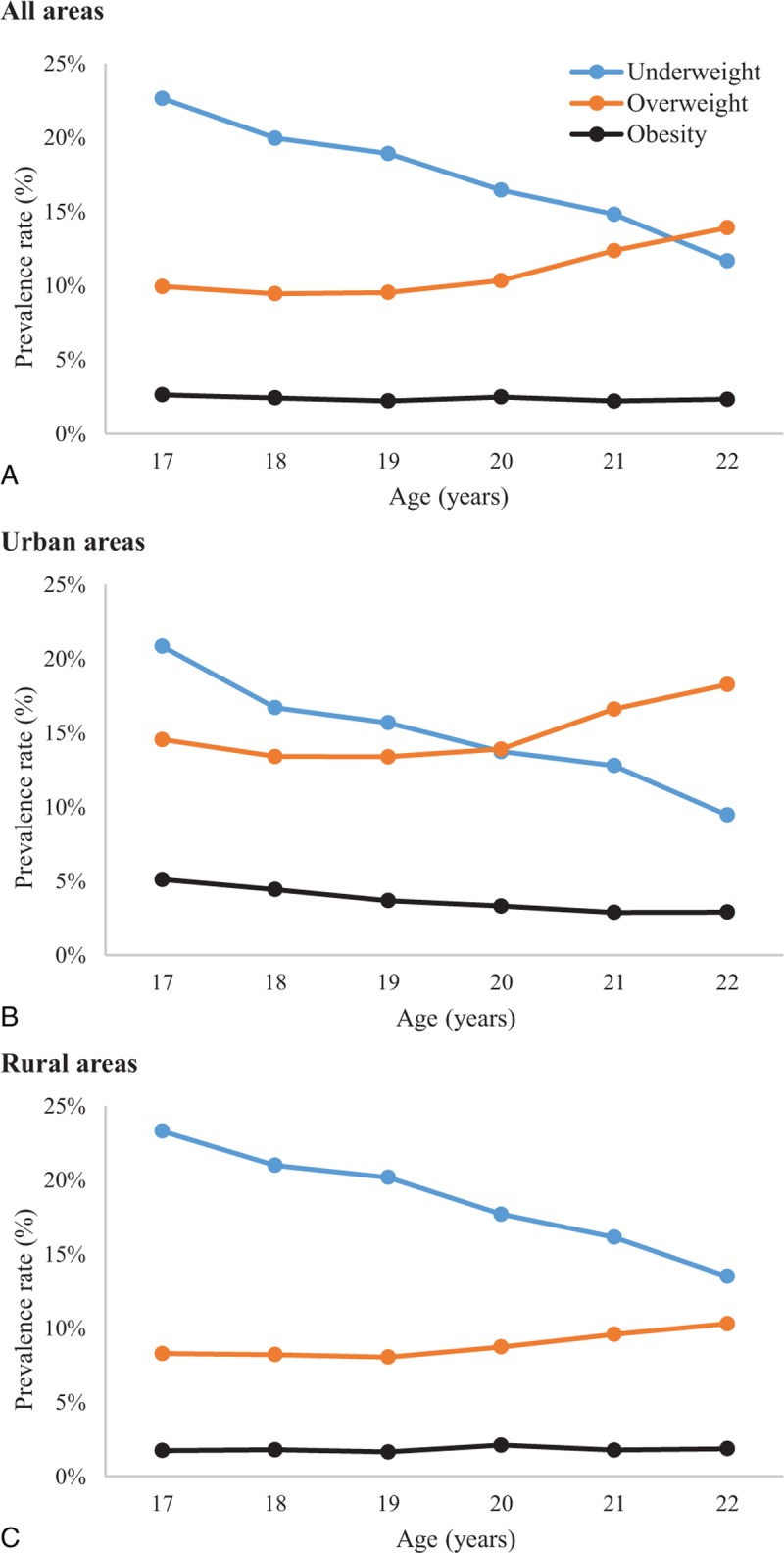
Relationships between age and prevalence of underweight, overweight, and obesity in 2012.

Data from the 2012 national survey showed that age was correlated positively with the prevalence of overweight and negatively with the prevalence of underweight (both *P* < 0.05). The prevalence of obesity showed a stable trend across ages. Furthermore, the prevalence of underweight, overweight, and obesity differed significantly between urban and rural areas (all *P* < 0.05). The trend lines of decreasing underweight prevalence and increasing overweight prevalence crossed at the age of 20 years for urban areas, but not until after the age of 22 years for rural areas (Figure 3).

**FIGURE 4:**
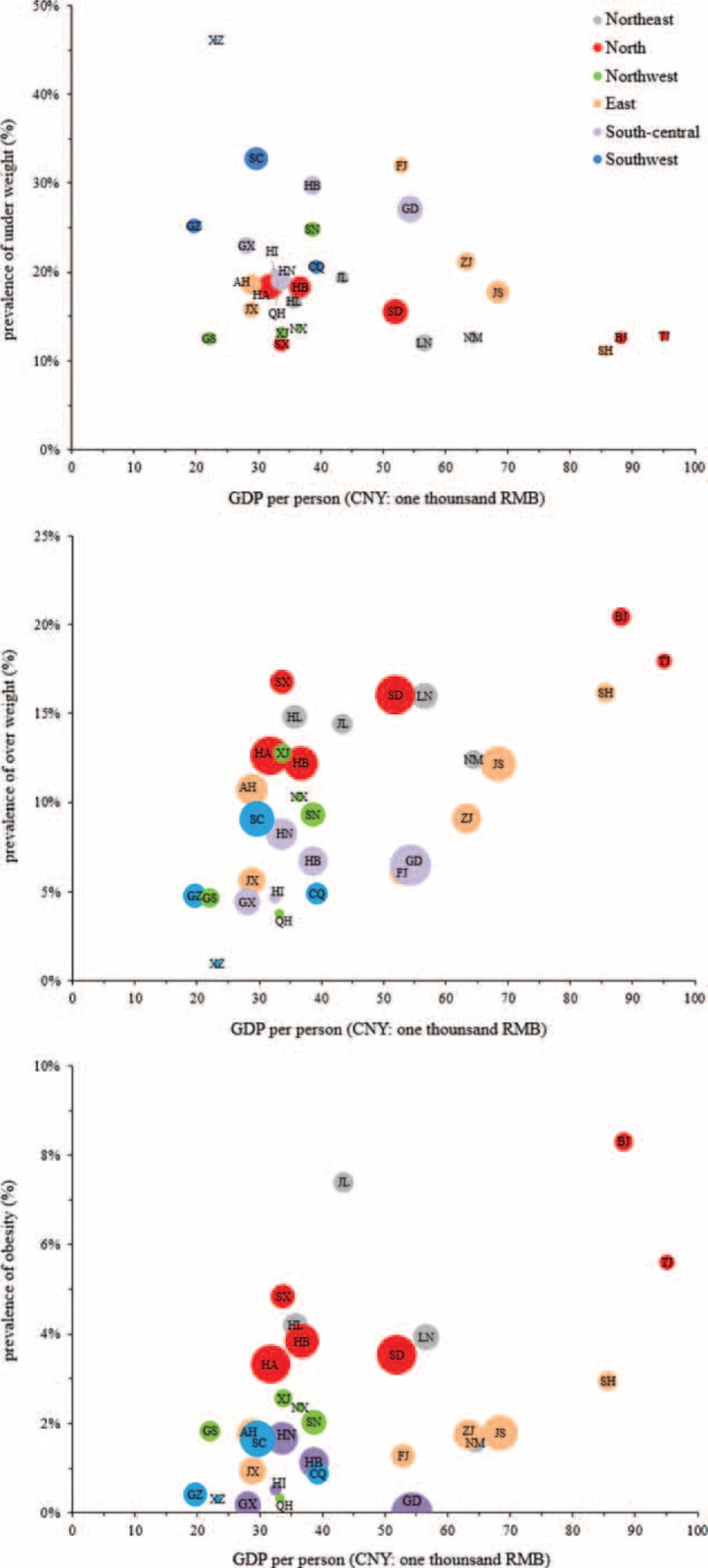
Relationships between GDP and prevalence of underweight, overweight, and obesity in 2012. Correlation coefficients between GDP and the prevalence of underweight, overweight, and obesity (weighted by population average) are *r* = –0.247, 0.446, and 0.240, respectively (all *P* < 0.001). BJ = Beijing, CQ = Chongqing, FJ = Fujian, GD = Guangdong, GDP = gross domestic product, GS = Gansu, GX = Guangxi, GZ = Guizhou, HA = Henan, HB = Hubei, HE = Hebei, HI = Hainan, HL = Heilongjiang, HN = Hunan, JL = Jilin, JS = Jiangsu, JX = Jiangxi, LN = Liaoning, NM = Inner Mongolia, NX = Ningxia, QH = Qinghai, SC = Sichuan, SD = Shandong, SH = Shanghai, SN = Shaanxi, SX = Shanxi, TJ = Tianjin, XJ = Xinjiang, XZ = Tibet, YN = Yunnan, ZJ = Zhejiang.

As the data clearly showed geographic differences in BMI across China, relationships between the prevalence of underweight, overweight, and obesity in men aged 18 to 20 years and the GDPs of provinces, autonomous regions, and municipalities in 2012 were further analyzed. Analysis weighted by the population average revealed a negative correlation between GDP and the prevalence of underweight (*r* = –0.247) and positive correlations between GDP and the prevalence of overweight and obesity (*r* = 0.446 and 0.240, respectively; all *P* < 0.001; Figure 4). Tibet (XZ) had the highest prevalence of underweight and the lowest prevalence of overweight, with a low per-person GDP. Furthermore, provinces and municipalities with high per-person GDPs, such as Beijing (BJ) and Tianjing (TJ), had the lowest prevalence of underweight and the highest prevalence of overweight or obesity.

## DISCUSSION

The results of this study showed increasing trends in BMI of men aged 18 to 20 years from 1955 to 2012, at the national level and in each of the 6 areas of China. Furthermore, BMI was found to differ among geographic regions of China. Analysis of 2012 national survey data showed that age (17–22 years) was positively correlated with the prevalence of overweight and negatively correlated with the prevalence of underweight. GDP was negatively correlated with the prevalence of underweight and positively correlated with the prevalence of overweight and obesity.

Overweight and obesity is a major public health challenge in many countries, and tracking of this important health risk with increased precision and disaggregation is a key global health priority.^11^ Studies have shown geographic differences and an overall increasing trend in BMI in most countries,^16,21,22^ similar to our results. As shown in Figure 1, from 1955 to 2012 the national average annualized rate of BMI increased with acceleration in recent years. Furthermore, compared with other regions in China, BMI and the prevalence of overweight and obesity were higher, and the prevalence of underweight was lower, among young men in northern China.

The study results showed marked variation in the prevalence and trends of overweight and underweight between urban and rural areas, and heterogeneity among urban sites. Furthermore, the results confirmed the existence of relationships between GDP and underweight, overweight, and obesity across the 30 provinces, autonomous regions, and municipalities in China. Two or 3 generations ago, China faced a nutritional challenge, with many people suffering from malnutrition and underweight.^14,23,24^ However, the opposite problem is emerging rapidly, especially in large cities. With increased quality of living conditions, overweight and obesity are becoming more prevalent. In contrast to the U.S., where obesity is concentrated among poorer people, China's obesity problem is largely defined by what the French has termed a “wealth-deficit” problem.^19^ With China's meteoric economic rise and fast-food market growth, lifestyles have changed dramatically. To counter the impending health effects on populations, measures are needed to effectively intervene against major determinants, such as excessive caloric intake, physical inactivity, and active promotion of food consumption by industry, all of which exacerbate an already problematic obesogenic environment.

The study results showed the common phenomenon of the coexistence of underweight and overweight in China, with underweight being slightly more prevalent, as in many other developing countries.^21,25–27^ Although overweight has become a problem, especially in large cities with developed economies, the national prevalence of overweight and obesity among young men remains low compared with other developed countries. Although China has shown great improvement in diet and nutritional status for youths, underweight remains an important nutritional problem, especially among youths in rural areas and western regions. The coexistence of underweight and overweight could also be explained by the pronounced diversity in economic status and development levels among areas of China. With the increasing investment of the Chinese government in western regions and improvements in the balance of development, further measures should be taken to address the problems of underweight and overweight, and complex measurement strategies need to be taken into account as options for population-level surveillance of these epidemics.

This study has several limitations. First, study data were collected from 4 national surveys conducted between 1955 and 2012, and many factors influencing survey results might not be exactly the same over this long-time span. However, all 4 surveys were conducted by the same leading institute, with a stable academic organization. Furthermore, the survey design and procedure, including sampling method, outcome measurement, and data collection, was kept consistent to the greatest extent possible overtime, facilitating the comparison of results. Second, as only statistical analysis results, not raw data, were available for the 1955 and 1974 surveys, the statistical estimation of BMI may have produced results that deviate slightly from the real values. However, the estimation was based on a large sample and this potential deviation is small enough to be ignored; hence, the results can be considered acceptable for use in the present study. Third, the study included only participants of Han nationality, and the results do not reflect BMI status among Chinese people of other nationalities. The Han nationality is the largest of 56 nationalities in China, accounting for 91.02% of the country's population.^28^ This study focused on the Han population because the limited numbers of young men of minority nationalities and the limited geographic distributions of these groups might increase bias and affect comparisons. Finally, although BMI is a convenient measure of adiposity, it does not adequately account for variation in body structure among ethnic groups.^29^ Although the use of internationally accepted BMI cut-off values did not affect internal comparison results in this study, it may have led to underestimation of the actual prevalence of overweight and obesity in China.

## CONCLUSION

BMI increased with economic development among young men from 1955 to 2012, with distinct variation among geographic areas in China. Although underweight remains prevalent in young men, especially in urban and northern regions, overweight and obesity are increasingly prevalent and warrant public health attention. Further longitudinal studies of the effects of BMI and physical status on disease prevention and control in diverse populations are recommended.
